# Commercial determinants of youth smoking in ASEAN countries: A narrative review of research investigating the influence of tobacco advertising, promotion, and sponsorship

**DOI:** 10.18332/tid/139124

**Published:** 2021-07-20

**Authors:** Thomas Stubbs

**Affiliations:** 1 School of Psychology, Faculty of Health, Deakin University, Burwood, Australia

**Keywords:** tobacco, smoking, youth, TAPS, ASEAN

## Abstract

**INTRODUCTION:**

Tobacco smoking is one of the leading causes of death and disability in the Association of Southeast Asian Nations (ASEAN). Despite implementation of some tobacco control measures, youth continue to initiate smoking. This narrative review outlines how tobacco advertising, promotion, and sponsorship (TAPS) may influence smoking attitudes and uptake among youth in the region.

**METHODS:**

Nine electronic databases were searched on EBSCOhost to identify studies published up until December 2019. All studies published in English that investigated youth smoking and TAPS in ASEAN countries were included. Thematic analysis was used to investigate the influence of TAPS on youth smoking.

**RESULTS:**

Thirty-seven studies were identified. This research showed that youth were exposed and receptive to tobacco advertising, which may contribute to positive attitudes towards tobacco brands and smoking. Studies also demonstrated that youth were exposed to point-of-sale (POS) advertisements or promotions and individual sales promotions. However, little research has explored how these strategies influence attitudes and consumption behaviors among youth, or, how online advertising and cigarette packet branding may influence youth smoking.

**CONCLUSIONS:**

Youth in ASEAN countries continue to be exposed to TAPS, particularly through POS advertisements or promotions and individual sales promotions. There is also cause for concern about ‘below-the-line’ advertising and the increasing role of cigarette packaging as a promotional tool. These findings support calls for all ASEAN countries to ratify the Framework Convention on Tobacco Control (FCTC), introduce comprehensive bans on all forms of tobacco advertising, including POS advertising and cigarette pack displays, and implement plain packaging legislation for tobacco products.

## INTRODUCTION

The World Health Organization’s (WHO) Global Action Plan for the Prevention and Control of Noncommunicable Diseases 2013–2020 calls for a 30% reduction in the prevalence of tobacco use among people aged ≥15 years by 2025^1^. However, neither the Southeast Asian nor Western Pacific region are projected to reach this target^2^. These regions comprise the ten countries that make up the Association of Southeast Asian Nations (ASEAN): Cambodia, Lao People’s Democratic Republic (Lao PDR), Myanmar, Vietnam, Indonesia, the Philippines, Thailand, Malaysia, Brunei Darussalam, and Singapore^3^. The ASEAN region is home to approximately 10% of the world’s one billion smokers^4^. The range of noncommunicable diseases and economic costs associated with tobacco use are a major burden to developing countries throughout this region^5-10^. One factor contributing to this issue is the continued uptake of smoking among youth, defined by the United Nations as young people aged 15–24 years^11^. Studies show that smokers in ASEAN countries often start using tobacco during the critical developmental period between adolescence and early adulthood^12-14^, which has far-reaching implications for their future health and wellbeing^15^.

Commercial determinants of health, of which marketing is a key pillar, are defined as the ‘strategies and approaches used by the private sector to promote products and choices that are detrimental to health’^16^. Tobacco advertising, promotion, and sponsorship (TAPS) have been used by the tobacco industry to increase demand for their products, often through targeting different groups or market segments^17^. Analysis of tobacco industry documents have highlighted that young people are a crucial market for tobacco companies^18^, who use TAPS to create brand associations that appeal to youth or establish brand loyalty among current smokers^19^. Research has demonstrated that tobacco advertising targets the psychological and social desires of youth, such as peer approval, maturity, fashion, masculinity, and femininity^20,21^. Tobacco companies have used a range of marketing strategies to communicate brand associations to youth, including paid advertisements on billboards, television, and radio^19^, as well as emerging technologies on websites and social media^22-25^.

Research from high-income countries has demonstrated that TAPS influence youth’s attitudes and behaviors around smoking. For example, studies have shown that exposure to tobacco advertising was associated with increased smoking uptake among youth^26-31^, while receptivity to tobacco advertising was associated with increased smoking susceptibility and uptake^32,33^. Evidence also suggests that tobacco promotions may influence smoking attitudes and consumption behaviors among youth^34,35^. These strategies include point-of-sale (POS) advertisements or promotions to increase the visibility of tobacco products to potential consumers, to trigger impulse buying among current or former smokers, or to normalize tobacco products by positioning them alongside everyday items in retail stores^36,37^. Cigarette packaging is also crucial to the tobacco industry’s promotional strategy, which includes brand names, colors, logos, slogans, product descriptions, and images^38^. Research has demonstrated that cigarette pack colors and product descriptions influence youth’s beliefs about the perceived harm^39,40^ and the taste and appeal of tobacco products^41,42^. Studies also suggest that cigarette packaging may influence their attitudes towards tobacco brands, including their beliefs and stereotypes about the typical smoker of different brands^43,44^.

ASEAN countries have implemented various policies to restrict TAPS. Except for Indonesia, all countries have ratified the WHO Framework Convention on Tobacco Control (FCTC)^4^. However, implementation of FCTC Articles differs across the region in terms of their coverage, implementation, and enforcement. While all ASEAN countries have implemented some bans on the direct advertising of tobacco products, partial bans in some countries, such as the Philippines and Indonesia, still permit indirect advertising^3^. Moreover, POS advertisements, cigarette pack displays, and individual sales promotions are still permitted in several countries across the region^4^. Evidence also suggests that lack of enforcement of TAPS restrictions remains an issue across the region, particularly for internet and social media advertising^3^. Despite the introduction of these TAPS restrictions across the region, recent evidence shows that youth from all ASEAN countries have continued to be exposed to various advertising and promotional strategies^4^.

Given that smokers in ASEAN countries often start using tobacco during adolescence and early adulthood^12-14^, this study aimed to review evidence related to how TAPS might influence the smoking attitudes and behaviors of youth in ASEAN countries. Findings will help to identify gaps in knowledge and provide recommendations for future research and public health policies.

## METHODS

### Search strategy

A search strategy was developed to identify relevant studies published from January 1999 to December 2019 (Table 1). This included a combined search of four terms related to youth smoking in ASEAN countries (‘smoking’ AND ‘ASEAN’ AND ‘youth’ AND ‘advertising’). This search was designed to capture possible variations of each search term (e.g. ‘smoking’ OR ‘smoke’). Using EBSCOhost, nine electronic databases were searched: Business Source Complete; CINAHL Complete; Global Health; Health Policy Reference Center; Health Source Consumer Edition; Health Source Nursing/Academic Edition; MEDLINE Complete; PsycINFO; and SocINDEX. These databases were chosen based on their relevance to one or more of the search terms included in the search strategy. The search criteria on EBSCOhost were limited to studies that were published in English, so that studies published in other languages were excluded from the search results. To identify any missed studies, the reference lists of relevant studies were reviewed and Google Scholar was searched using the terms ‘youth smoking and [country name]’, with the first five pages of results reviewed. This search took place between December 2019 and February 2020.

**Table 1:** Search strategy to identify studies relating to TAPS and youth smoking in ASEAN countries

*Search terms*	*Variations*
Term 1	‘smoking’ OR ‘smoke’ OR ‘smoker*’ OR ‘tobacco’ OR ‘cigarette*’ OR ‘nicotine’
Term 2	‘ASEAN’ OR ‘Asia’ OR ‘Asian’ OR ‘Cambodia*’ OR ‘People's Democratic Republic of Lao’ OR ‘Lao PDR’ OR ‘Lao’ OR ‘Laos’ OR ‘Myanmar’ OR ‘Vietnam*’ OR ‘Viet Nam*’ OR ‘Indonesia*’ OR ‘Philippines’ OR ‘Filipino*’ OR ‘Thailand’ OR ‘Thai’ OR ‘Malaysia*’ OR ‘Malay’ OR ‘Brunei’ or ‘Brunei Darussalam’ OR ‘Singapore’ OR ‘Singaporean’
Term 3	‘youth*’ OR ‘adolescent*’ OR ‘adolescence’ OR ‘young people’ OR ‘teen*’ OR ‘young adults’ OR ‘children’ OR ‘kids’
Term 4	‘advertising’ OR ‘advertisement*’ OR ‘ads’ OR ‘commercial*’ OR ‘promotion*’ OR ‘sponsorship*’ OR ‘POS’ OR ‘point of sale’ OR ‘TAPS’ OR ‘marketing’ OR ‘branding’ OR ‘brand*’ OR ‘media’ OR ‘online’ OR ‘website*’ OR ‘internet’ OR ‘social media’ OR ‘Facebook’ OR ‘Twitter’ OR ‘Instagram’

### Inclusion criteria

Studies were included if they related to TAPS, were conducted in ASEAN countries, and included participants aged 13–24 years. This age group was selected to identify research relating to smoking initiation during early adolescence to early adulthood, which is when most smokers in the region take up tobacco use^12-14^.

### Screening process

**Figure 1:**
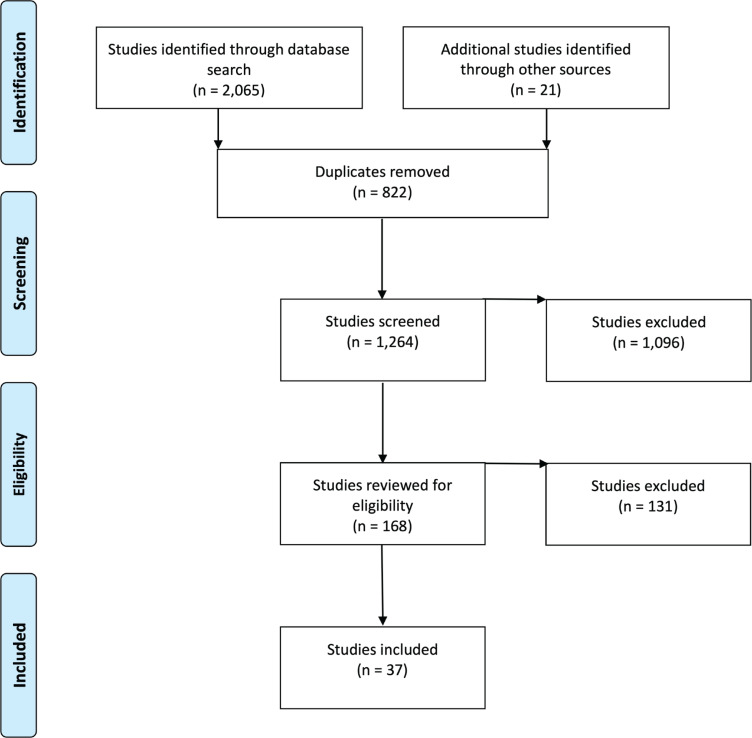
PRISMA flow diagram for selection of studies relating to TAPS and youth smoking in ASEAN countries

Figure 1 illustrates the inclusion and selection process for this review. The initial database search yielded 2065 results, with 21 studies added from searching Google Scholar and the reference lists of relevant studies. Search results were combined, and 822 duplicates were removed. An initial assessment included reading the title of the remaining 1264 studies, which resulted in 1096 being excluded. Abstracts of the remaining 168 studies were then read, with 37 articles meeting the criteria for the review. Studies were excluded because they were conducted in countries outside ASEAN countries, did not focus on youth, or were concerned with other forms of tobacco use. The quality of all 37 articles was then assessed using checklists from the Critical Appraisal Skills Programme, with each study assessed using the appropriate checklist for its method^45^. This revealed that all 37 articles met a minimum standard for inclusion in the review. Findings from each study were then inserted into an Excel file for analysis.

### Analysis

Thematic analysis was performed on each of the studies. Findings were grouped into four themes: 1) advertising, 2) POS advertisements or promotions, 3) promotional strategies, and 4) sponsorship.

These themes were based on categories of different TAPS strategies reported in the literature^17^. The following definitions were used to distinguish between the different TAPS strategies: 1) advertising included mass media marketing using television, radio, print media, billboards, the internet, and tobacco imagery in the media; 2) POS advertisements or promotions included marketing in retail stores such as posters, coupons, or tobacco product displays; 3) promotional strategies included individual sales promotions and the distribution of free cigarettes or branded merchandise; and 4) sponsorship included paid endorsements, including naming rights, events, teams, individuals or organizations^17^.

## RESULTS

### Characteristics of included research articles

Supplementary file Table S1 provides a description of the studies included in this narrative review. A total of 25 studies were published in peer-reviewed journals and 12 articles in grey literature. Thirty-three studies used a quantitative design, three were qualitative, and one used a mixed-method approach. The studies included in the review were predominantly conducted in Indonesia (n=9), Vietnam (n=8), Thailand (n=6), and the Philippines (n=6); with fewer studies conducted in Malaysia (n=5), Myanmar (n=5), Cambodia (n=4), Lao PDR (n=4), Singapore (n=1), and Brunei Darussalam (n=1). This sample included some studies that were conducted across multiple countries, which is why the numbers above add up to greater than 37.

### Advertising

Studies in this review indicated that school children were exposed to tobacco advertising in ASEAN countries. However, this varied across different countries in the region. In Indonesia, 92.9% of school students (13–15 years) reported seeing advertisements for cigarettes on billboards^46^, with similar rates observed in the Philippines (87.6%)^47^ and Cambodia (85.1%)^48^. However, exposure to tobacco advertising was much lower among school children in Thailand (33.8%)^49^. Research also showed that school children were exposed to tobacco advertisements in outdoor settings, with 67.2% of school children in Myanmar and 74.1% of school children in the Philippines having seen tobacco advertisements at sporting, musical, and community events, or cultural festivals^50^. Recent studies also showed that school children were exposed to images of people using tobacco in the media. In 2016, one study identified that 83.4% of school children in Myanmar had seen people using tobacco on television, videos, or in movies^51^. Similarly, studies identified that school children had been exposed to tobacco imagery in Lao PDR (61.9%)^52^, Indonesia (62.7%)^53^, Cambodia (65.0%)^54^, the Philippines (70.1%)^55^, Thailand (77.4%)^56^, and Vietnam (77.8%)^57^. However, these studies did not report whether the tobacco images were part of a tobacco advertisement or general tobacco use. Moreover, the prevalence of exposure reported in these studies only related to school children who had watched television, videos, or movies in the past 30 days.

Several studies indicated that tobacco advertising may influence smoking susceptibility and consumption among youth in ASEAN countries. In Vietnam, researchers identified that exposure to tobacco advertising was associated with an increased risk of smoking among school children (13–15 years)^58^. This association was also observed in several Indonesian studies, where exposure to tobacco advertising was associated with current smoking among school students^59,60^ and youth (11–24 years)^61^. In another Indonesian study, researchers demonstrated that a higher level of exposure to tobacco advertising was associated with increased smoking among adolescent smokers^62^. Another study suggested that tobacco advertising may influence positive attitudes among non-smoking youth in Vietnam, with exposure to cigarette advertising associated with increased smoking susceptibility among female school students (13–15 years)^63^.

Studies in this review demonstrate that certain population groups respond differently to tobacco advertising, with some groups being more vulnerable to tobacco advertising than others. Based on data from the Global Youth Tobacco Survey (GYTS) conducted in Vietnam, Laos, and Cambodia, research identified that exposure to tobacco advertising was a risk factor for susceptibility to smoking initiation among school girls, but not school boys^64^. However, a study in Vietnam showed that recall of tobacco advertising was more likely among adolescent males than females^65^. Another important difference that was identified through the review was that youth may be at an increased risk of exposure to tobacco advertising or promotions than adults^66,67^. However, no studies provided an explanation for these differences.

Research suggests that youth in ASEAN countries were receptive to tobacco advertising. In Lao PDR, recognition of tobacco advertising was associated with smoking susceptibility among non-smoking, male school students (12–19 years)^68^. In Indonesia, having a positive attitude towards tobacco advertising was associated with an increased risk of smoking initiation and current smoking among teenagers (13–18 years)^59^. In the Philippines, drawing on data from the GYTS, research found that school children who smoked Marlboro cigarettes were more likely than smokers of other brands to think that boys who smoked were ‘macho’ and girls who smoked were ‘glamorous’^69^. This study also showed that smokers of Marlboro cigarettes were more likely than smokers of other brands to have intentions to smoke in the future, believe that smokers have more friends, or think that smoking helps to feel comfortable in social settings^69^.

The mixed-method and qualitative studies in this review explored how tobacco advertising may influence youth’s attitudes towards tobacco brands and smoking. In Indonesia, research showed that teenage boys associated tobacco brands with positive attributes around gender norms and masculinity – such as independence or maturity^70^. A Malaysian study suggested that youth may find tobacco advertising appealing, describing imagery in cigarettes advertisements with appealing attributes such as maturity, style, masculinity, and attraction to the opposite sex^71^. A study in the Philippines also suggested that tobacco advertising might influence youth to form beliefs about the typical smokers of different cigarettes brands, with youth stating that Marlboro cigarettes were attractive because they promoted ‘adventure’ and ‘freedom’, whereas a local brand was perceived as more suitable for older smokers^72^. Conversely, young female smokers (14–21 years) in Malaysia reported that smoking imagery in the media did not influence them to start smoking, although it did encourage them to continue smoking if they had already adopted the behavior^73^.

Research regarding online tobacco advertising and youth in ASEAN countries is sparse, with studies predominantly focused on reporting exposure to this form of advertising among school children. GYTS data indicated that 22.1% of school children in Vietnam in 2014^65^ and 38.1% of school children in Thailand in 2015^74^ recalled being exposed to online tobacco advertising in the past 30 days. The Thai study also identified that online advertising was associated with an increased risk of smoking among school children (13–15 years)^74^. However, no studies in the review explored the impact of online tobacco advertising on smoking attitudes or the specific online platforms where youth recalled these advertisements.

### POS advertisements or promotions

Studies concerning POS advertising or promotions and youth in ASEAN countries mostly focused on measuring exposure to this form of marketing. Data from the GYTS showed that youth were exposed to POS advertising or promotions across the region, with school children (13–15 years) recalling seeing this marketing tool in Brunei Darussalam (27.6%)^75^, Vietnam (27.9%)^57^, the Philippines (50.6%)^55^, Thailand (35.5%)^56^, Lao PDR (35.2%)^52^, and Myanmar (42.3%)^51^. Recent studies identified that exposure to POS advertisements or promotions varied across different countries in the region, with one study showing that 17.7% of school children in Cambodia had noticed POS advertisements or promotions^54^, while another showed that 60.7% of school children in Indonesia had noticed this strategy^53^.

Similar to advertising, studies indicate that certain populations may be more vulnerable to POS advertisements or promotions. One study showed that youth (15–24 years) in Thailand were more likely than adults (≥25 years) to notice tobacco promotions in retail stores^66^. Research also identified that school boys (47.3%) were more likely than school girls (37.0%) to notice POS advertisements or promotions in Myanmar^76^. Two studies investigated the impact of POS advertisements or promotions on youth smoking attitudes and behaviors. These studies demonstrated that exposure to POS advertisements or promotions was associated with increased risk of smoking among school children (13–15 years) in Thailand^74^, while noticing POS advertisements or promotions was associated with increased susceptibility to smoking initiation among non-smoking adolescents (12–19 years) in Malaysia^77^.

### Promotional strategies

Several studies explored the influence of individual sales promotions and the distribution of free cigarettes or branded merchandise among youth in ASEAN countries. GYTS data indicated that school children had been exposed to this form of promotion, with 9.0% of school children in the Philippines reporting that they had been offered a free tobacco product from a tobacco company representative^55^. School children had also been offered a free tobacco product from a tobacco company representative in Myanmar (5.9%)^51^, Indonesia (7.9%)^53^, Thailand (7.3%)^56^, Malaysia (5.0%)^78^, and Brunei Darussalam (5.5%)^75^. One Cambodian study showed that older youth (15–24 years) had also been exposed to this form of advertising, with 7.6% reporting being offered free samples of tobacco products in the past 30 days^79^. A limited number of studies have explored how this promotional strategy might influence youth smoking. Some studies demonstrated that being offered free cigarettes or merchandise by a tobacco company representative was associated with an increased risk of smoking among school children in Thailand^74,80^, while owning tobacco-branded merchandise was associated with smoking among school students (15–19 years) in Indonesia^81^. However, no studies investigated how this promotional strategy might influence their attitudes towards smoking or tobacco brands.

### Sponsorship

Research on tobacco sponsorship and youth smoking in ASEAN countries is limited. Three studies included in this review showed that exposure to this advertising strategy varied across different countries in the region, with one identifying that only 1.6% of youth (15–24 years) in Vietnam recalled seeing tobacco sponsorship in the past 30 days in 2010^82^, while 7% of youth (15– 24 years) in Cambodia recalled tobacco sponsorship in 2011^79^. In Lao PDR, however, one study showed that 42.2% of non-smoking male students (12–19 years) noticed this form of advertising at sports or community events in 2010^68^. No studies in the review explored how tobacco sponsorship might influence smoking attitudes and behaviors among youth in the region.

## DISCUSSION

This study reviewed evidence related to how TAPS may influence the smoking attitudes and behaviors of youth in ASEAN countries. Studies clearly indicated that youth in the region were exposed to tobacco advertising^46-57^, and that exposure was associated with increased risk of smoking susceptibility or uptake^58-61^, as well as increased cigarette consumption among current smokers^62^. While quantitative studies suggested that school children in ASEAN countries were susceptible to tobacco advertising^68,69^, qualitative and mixed-method studies demonstrated that tobacco advertising might influence youth to form positive attitudes towards smoking and tobacco brands – particularly around gender norms and smoking^70-72^. This may suggest a gender bias underpinning tobacco smoking in ASEAN countries. However, further research is needed to explore how sociocultural factors and tobacco advertising may influence smoking attitudes and behaviors among youth.

Studies showed that school children in ASEAN countries were exposed to online tobacco advertising^49,65^, and that exposure to this form of advertising increased their risk of smoking uptake^74^. However, the scope of these studies was limited and did not identify where youth recalled seeing tobacco advertising, such as particular online platforms, their perceptions of these advertisements, or how online advertising might impact their beliefs and attitudes towards tobacco brands or smoking. This lack of research on online tobacco advertising and youth smoking in ASEAN countries is an important gap identified in this review, particularly with the increasing use of the internet and social media observed across the region^83^. Recent research also suggests the tobacco industry is utilizing sophisticated online strategies to promote their brands to youth in the region. For example, Astuti et al.^84^ note that a tobacco company in Indonesia used music event sponsorship and Instagram to appeal to youth^84^. The event included a tobacco-branded photobooth and social media hashtag that encouraged young concert goers to capture and share their experiences online with friends^84^. Although this research suggests that tobacco companies may be using online strategies to target youth or complement other forms of TAPS, limited research in this review explored how these engaging and personalized strategies may influence young people’s attitudes towards tobacco brands and smoking.

The studies reviewed indicated that school children in ASEAN countries were exposed to POS advertisements or promotions^51-57^ and that exposure to these was associated with an increased risk of smoking susceptibility^77^. However, there is currently limited research from ASEAN countries to explain how exposure to POS advertisements or promotions shape their attitudes towards cigarette brands or smoking. This is an important gap in the literature given that tobacco companies have used this strategy to promote their brands^36,37^. Moreover, evidence suggests that online and social media technologies are being used to augment POS advertisements or promotions in the ASEAN region. A recent study showed that a tobacco company in Indonesia displayed social media hashtags and website links alongside POS cigarette pack displays and promotions, which linked users to youthfocused content and online communities sponsored by the tobacco industry^85^. However, further research is needed to explore how youth engage with these augmented promotions, or how this may influence their smoking attitudes and behaviors.

The review has highlighted that youth have been offered free tobacco products in ASEAN countries^53-55^, and that exposure to this promotional strategy was associated with an increased risk of smoking^74,80^. These findings align with previous research which shows that tobacco companies have distributed free cigarette samples or tobacco-branded merchandise in the region^86^, despite bans on this promotional strategy in almost all countries in the region^3^. However, it is currently unknown in what contexts these individual sales promotions are being carried out, or how these promotions may influence young people’s willingness to try smoking or attitudes towards tobacco brands.

Although research indicates that cigarette pack branding influences the smoking beliefs and attitudes among youth in Western countries^39-44^, studies from ASEAN countries appear to be absent. This is an important gap in the literature as product packaging has become one of the primary advertising strategies that tobacco companies use to communicate brand imagery^38^. Consequently, future studies in ASEAN countries could explore how cigarette packet elements might shape young people’s attitudes towards tobacco brands and smoking. Studies could also explore how youth perceive plain packaging compared to branded packaging, or how plain packaging might challenge their current attitudes towards cigarette brands or smoking.

The research shows that youth in ASEAN countries were exposed to tobacco sponsorship^68,79,82^. However, this finding should be considered in light of two important limitations. First, it was unclear whether participants in these studies distinguished between tobacco advertising in outdoor settings, such as sports events or festivals, and tobacco sponsorship. It is, therefore, possible that participants conflated the two strategies when recalling their exposure of the two strategies. Second, use of this strategy may have decreased in recent years with the introduction of comprehensive or partial bans on tobacco sponsorship across the region^3^. Nonetheless, further research could uncover ways in which tobacco companies might still be using sponsorship to target youth in the region, or whether sponsorship is being used alongside other strategies, such as online and social media promotions, to increase brand appeal and engagement of youth^84^.

### Implications for policy

This review has implications for TAPS restrictions across the region. Although nine out of the ten ASEAN countries have ratified the FCTC and all countries have implemented some bans on the direct advertising of tobacco products^3^, several countries still permit some form of advertising. For example, Indonesia has only implemented a partial ban on tobacco advertising, with advertisements still permitted on television during specific hours in the evening and limited restrictions on the type of imagery or size of print and outdoor advertising, while in the Philippines, tobacco advertising is still allowed on video, audiocassettes, and in videogames to persons over 18 years old^3^. Closing these advertising channels is important for preventing exposure to TAPS among youth in ASEAN countries.

While most ASEAN countries have introduced restrictions on POS advertisements or promotions, in line with Article 13 of the FCTC, Indonesia and the Philippines still permit tobacco companies to advertise their products at POS^3^. Moreover, the current legislation in Cambodia still allows retailers to display the logo or name of tobacco products at POS and to show one cigarette pack of each brand sold^3^. These partial restrictions still enable tobacco companies to maintain visibility of their products and brands, and highlight the importance of implementing complete bans on POS advertisements or promotions^17^. Thailand addressed this issue through a complete ban on the display of tobacco products at the POS, which was recently followed by Brunei and Singapore which have introduced similar restrictions^3^. All ASEAN countries should introduce complete bans on POS advertisements or promotions, including cigarette pack displays, to prevent exposure to these marketing strategies among youth.

Under Article 11 of the FCTC, signatories should implement legislation requiring tobacco products to display pictorial health warnings, which communicate the harms associated with tobacco use^87^. Alongside this, they should also introduce plain packaging legislation that prohibits the use of logos, colors, brand imagery, or promotional information on tobacco products, with brand names to be displayed in a standard color and font^87^. While all ASEAN countries require tobacco product packaging to display pictorial health warnings^4^, Thailand and Singapore are the only countries in the region to adopt plain packaging legislation^88^. Although research on cigarette pack branding and youth is limited in the region, studies from western countries provide sufficient evidence to support urgent calls for introducing plain packaging laws in ASEAN countries^39-44^.

### Limitations

This review must be considered within the context of several limitations. Because selection criteria limited studies to those published in English, studies presented in other languages were not included in this study. With the search strategy predominantly focused on peer-reviewed journal articles, it is also possible that relevant grey literature may have been missed in the search process. Some research included in the review was conducted more than 10 years ago, which limits its compatibility to more recent research, and may not reflect exposure to TAPS in countries that have implemented restrictions in recent years. However, these studies still provide important insights on the TAPS situation in ASEAN countries, as recent research suggests that young people continue to be exposed to tobacco advertising and promotional strategies throughout the region^4^, as well as evidence that lack of implementation or enforcement of current restrictions remains an issue in some countries^3^. Finally, some of the research included in the review used data from the GYTS, which only included school children aged 13–15 years. This could mean the results may not be directly relevant for out-of-school children or youth in older age groups.

## CONCLUSIONS

Despite implementation of various Articles under the FCTC, ASEAN countries still face significant challenges from tobacco smoking. One factor hindering progress in reducing the prevalence of tobacco smoking may be the uptake of smoking among youth, who are particularly vulnerable to commercial factors that influence them to start smoking. Although most ASEAN countries have implemented comprehensive bans on TAPS, this review indicates that youth may still be exposed to more subtle forms of marketing, such as POS advertisements or promotions, individual sales promotions, and cigarette pack branding. Research also suggests that online and social media strategies are being used to market tobacco brands to youth, and potentially to augment other forms of TAPS. There is limited research into how these strategies might influence young people’s attitudes and behaviors around smoking, or how emerging strategies may be bypassing current TAPS restrictions across the region. Despite these gaps, this review supports calls for improved enforcement of current TAPS restrictions, expanding restrictions around POS advertisements or promotions, and introducing plain packaging laws to address the potential influence of cigarette pack branding on youth smoking.

## Data Availability

The data supporting this article can be found in the Supplementary file.
