# Supplementary material for: Commercial determinants of youth smoking in ASEAN countries: A narrative review of research investigating the influence of tobacco advertising, promotion, and sponsorship
Source: Tob Induc Dis. 2021 Jul 20;19:61. doi: 10.18332/tid/139124 (PMC8288466; doi:10.18332/tid/139124)
Supplement: Supplementary file 1 [file TID-19-61-s1.pdf]

Supplementary file 1: Details of studies included in narrative review on TAPS and youth smoking in ASEAN countries

**Author(s) and year**	**Publication type**	**Country**	**Year of data collection**	**Study method**	**Sample**	**Key findings**
Aditama et al. 2008	Peer-reviewed	Indonesia	2006	Quantitative	School children aged 13-15 years (n=4,636)	92.9% of participants had seen a lot of advertisements for cigarettes on billboards within the past month; 82.8% had seen a lot of advertisements for cigarettes in newspapers or in magazines; 10.8% had an object with a cigarette or tobacco company logo on it
Agaku et al. 2013	Peer-reviewed	Myanmar and Philippines	2007	Quantitative	School children aged 13-15 years in Myanmar (n=3,118) and the Philippines (n=5,919)	67.2% of participants in Myanmar and 79.4% of participants in the Philippines reported seeing tobacco advertisements at sports events, musical concerts, community events, or cultural festivals in the past 30 days
Al-Sadat and Binns 2008	Peer-reviewed	Malaysia	2005-2006	Qualitative	Young women aged 14-21 years (n=84)	Participants recalled four themes: influence of peers, influence of seeing parents smoking, belief that smoking can reduce stress, and smoking impresses others; participants recalled that smoking imagery in the media did not influence them to start smoking, but did encourage current smokers to progress to regular smokers

Bigwanto et al. 2017	Peer-reviewed	Indonesia	2014	Quantitative	School children aged 15-19 years (n=690)	Owning a tobacco-branded promotional item was associated with current cigarette smoking
Cambodian National Institute of Statistics 2011	Grey literature	Cambodia	2011	Quantitative	Nationally representative sample of Cambodian population aged 15 years and older (n=15,615)	7.6% of participants aged 15-24 years reported being offered free samples of tobacco products in the past 30 days; 7% recalled tobacco sponsorship in the past 30 days; 17.7% owned clothing or an item with tobacco brand name or logo
CDC 2005	Peer-reviewed	Philippines	2000 and 2003	Quantitative	School children aged 13-15 years, with surveys conducted in 2000 (n=11,630) and 2003 (n=7,478)	84.4% of participants in 2000 and 87.6% of participants in 2003 saw pro-tobacco message on billboards during the past 30 days; 80.4% of participants in 2000 and 81.7% of participants in 2003 saw pro-tobacco message in newspapers or magazines during the past 30 days; 17.8% of participants in 2000 and 16.4% of participants in 2003 had an object that features the logo of a tobacco company
CDC 2009	Grey literature	Malaysia	2009	Quantitative	School children aged 13-15 years (n=3,021)	5.0% of participants had been offered free cigarettes by a tobacco company representative; 9.2% of participants had an object with a cigarette brand logo

CDC 2010	Peer-reviewed	Thailand	2009	Quantitative	People aged 15 years and older in three countries, including Thailand (n=20,566)	Among participants aged 15–24 years, 25.1% of males and 28.1% of females noticed any cigarette advertising, sponsorship, or promotion; 12.1% of males and 15.2% of females were aware of tobacco marketing in stores where cigarettes are sold
CDC 2013	Grey literature	Brunei Darussalam	2013	Quantitative	School children aged 13-15 years (n=917)	27.6% of participants had noticed tobacco advertisements or promotions at POS; 5.5% had ever been offered a free tobacco product from a tobacco company representative
CDC 2014a	Grey literature	Indonesia	2014	Quantitative	School children aged 13-15 years (n=4,317)	60.7% of participants had noticed tobacco advertisements or promotions at POS; 62.7% had seen anyone using tobacco on television, videos, or movies; 7.9% had ever been offered a free tobacco product from a tobacco company representative
CDC 2014b	Grey literature	Vietnam	2014	Quantitative	School children aged 13-15 years (n=3,430)	27.9% of participants had noticed tobacco advertisements or promotions at POS; 77.8% had seen anyone using tobacco on television, videos, or movies; 3.1% had ever been offered a free tobacco product from a tobacco company representative

CDC 2015a	Grey literature	Philippines	2015	Quantitative	School children aged 13-15 years (n=5,885)	50.6% of participants had noticed tobacco advertisements or promotions at POS; 70.1% had seen anyone using tobacco on television, videos, or movies; 9.0% had ever been offered a free tobacco product from a tobacco company representative
CDC 2015b	Grey literature	Thailand	2015	Quantitative	School children aged 13-15 years (n=1,721)	35.5% of participants had noticed tobacco advertisements or promotions at POS; 77.4% had seen anyone using tobacco on television, videos, or movies; 7.3% had ever been offered a free tobacco product from a tobacco company representative
CDC 2016a	Grey literature	Cambodia	2016	Quantitative	School children aged 13-15 years (n=1,866)	17.7% of participants had noticed tobacco advertisements or promotions at POS; 65.0% had seen anyone using tobacco on television, videos, or movies; 8.0% had ever been offered a free tobacco product from a tobacco company representative
CDC 2016b	Grey literature	Lao PDR	2016	Quantitative	School children aged 13-15 years (n=3,930)	35.2% of participants had noticed tobacco advertisements or promotions at POS; 61.9% had seen anyone using tobacco on television, videos, or movies; 3.9% had ever been offered a free tobacco product from a tobacco company representative

CDC 2016c	Grey literature	Myanmar	2016	Quantitative	School children aged 13-15 years (n= 2,621)	42.3% of participants had noticed tobacco advertisements or promotions at POS; 83.4% had seen anyone using tobacco on television, videos, or movies; 5.9% had ever been offered a free tobacco product from a tobacco company representative
Chotbenjamaporn et al. 2017	Peer-reviewed	Thailand	2015	Quantitative	School children aged 13-15 years (n=1,721)	38.1% of participants had noticed advertisement of cigarettes on the internet or other online media; factors associated with increased risk of tobacco use among participants included with exposure to advertisement of cigarettes or other tobacco products on the internet, having an object with a cigarette brand or logo, being offered a free tobacco product by a tobacco company, and exposed to POS promotions
Ministry of Health of Viet Nam 2010	Grey literature	Vietnam	2010	Quantitative	Nationally representative sample of Vietnamese population aged 15 years and older (n=9,925)	Among participants aged 15-24 years, 1.1% had noticed tobacco marketing on the internet, 1.6% had noticed sports sponsorship, 1.0% had noticed free samples

Guindon, Georgiades, and Boyle 2008	Peer-reviewed	Cambodia, Lao PDR and Vietnam	2002 (Cambodia), 2003 (Lao PDR), 2003 (Vietnam)	Quantitative	School children aged 13-15 years in Cambodia (n=2,011), Loa PDR (n=9,720), and Vietnam (n=9,507)	Smoking susceptibility was associated with exposure tobacco advertising among female participants but not male participants
Huong et al. 2017	Peer-reviewed	Vietnam	2015	Quantitative	Nationally representative sample of Vietnamese population aged 15 years and older (n= 8,996)	Compared to older participants (25 years and older), younger participants (15-24 years) were at an increased risk of exposure to tobacco advertising or promotions
Kin and Lim 2003	Grey literature	Malaysia	2003	Qualitative	Adolescent smokers and non-smokers aged 13-17 years (n=96)	Participants were receptive to tobacco advertising, recalling tobacco advertisements that promoted smokers as mature, stylish, attractive, and masculine; participants also described popular cigarette brands as exclusive and better quality

Lagasse et al. 2018	Peer-reviewed	Philippines	2016	Mixed-method	Adolescents aged 13-17 years in Manila, Philippines (n=623)	Participants described Marlboro's advertising in the Philippines as appealing to youth, referring to advertisements as 'cool', 'refreshing', and 'adventurous', while advertisements of a local comparison brand were described as 'dull', 'simple' and 'old fashioned'
Lim et al. 2019	Peer-reviewed	Malaysia	2016	Quantitative	Non-smoking school children aged 12-19 years (n=11,246)	Smoking susceptibility was associated with exposure to and liking of POS advertising
Long et al. 2016	Peer-reviewed	Vietnam	2014	Quantitative	School children aged 13-15 years (n=3,430)	22.1% of participants recalled tobacco advertising on the internet; 19.2% of participants recalled tobacco advertising at shops; 11.5% of participants recalled tobacco advertising at social events such as sport or fashion events; participants were most likely to recall exposure to tobacco advertising or promotions if they were male, were susceptible to smoking, had parents who both smoke, or had friends who smoke

McKnight-Eily et al. 2010	Peer-reviewed	Thailand	2005	Quantitative	School children aged 13-15 years (n=19,874)	21.0% of current smokers and 7.0% of nonsmokers had been offered a free cigarette by a tobacco company representative; current smoking was associated with being offered a free cigarette by a tobacco company representative among both male and female participants
Van Minh et al. 2010	Peer-reviewed	Vietnam	2007	Quantitative	School girls aged 13-15 years (n=7,967)	Participants who were exposed to billboard cigarette advertising were more likely to be susceptible to smoking than those who had not be exposed
Van Minh et al. 2011	Peer-reviewed	Vietnam	2007	Quantitative	School children aged 13-15 years (n=15,610)	Exposure to tobacco advertising was associated with an increased risk of smoking among participants
Ng, Weinehall, and Ohman 2007	Peer-reviewed	Indonesia	2005	Qualitative	School boys aged 13-17 years (n=50)	Themes focused on the socio-cultural norms and values around smoking, reasons for smoking, and perceptions of health risk, addiction, and quitting; participants also described positive attitudes towards tobacco brands and smoking, which were rooted in gender norms around masculinity and social status

Page and West 2012	Peer-reviewed	Philippines	2000, 2003, 2007	Quantitative	School children aged 13-15 years (n=27,027)	Smokers of Marlboro cigarettes were more likely than smokers of other brands to think that boys who smoked were ‘macho’ and girls who smoked were ‘glamorous’; smokers of Marlboro cigarettes were also more likely to have intentions to smoke in the future, believe that smokers have more friends, and think that smoking helps to feel comfortable in social settings
Pandayu, Murti, and Pawito 2017	Peer-reviewed	Indonesia	2017	Quantitative	Smokers and nonsmokers aged 11-24 years (n=200)	Exposure to cigarette advertisements was associated with increased risk of current smoking
Panduwinata, Murti, and Pawito 2018	Peer-reviewed	Indonesia	2018	Quantitative	Male school students (n=200)	Smoking was associated with exposure to tobacco advertising in the media
Prabandari and Dewi 2016	Peer-reviewed	Indonesia	2010	Quantitative	School students aged 13-18 years (n=2,115)	Smoking initiation was associated with exposure to cigarette advertising and having a positive attitude towards cigarette advertising; current smoking was associated with exposure to cigarette advertising and having a positive attitude towards cigarette advertising

Purnaningrum, Joebagio, and Murti 2017	Peer-reviewed	Indonesia	2017	Quantitative	Adolescent smokers (n=100)	High cigarette smoking was associated with high exposure to tobacco advertising
Rudatsikira et al. 2008	Peer-reviewed	Thailand	2005	Quantitative	School children aged 13-15 years (n=18,368)	33.8% of participants had seen a cigarette brand name on TV in the past 30 days; 40.8% of participants had an item with a cigarette brand logo on it; 45.5% of participants had seen tobacco advertisements on the internet in the past 30 days, with males (48.4%) more likely than females (41.2%) to recall seeing tobacco advertisements online
Saito et al. 2013	Peer-reviewed	Lao PDR	2010	Quantitative	Nonsmoking, male school children aged 12-19 years (n=526)	Recognition of tobacco marketing messages was associated with smoking susceptibility; recognition of marketing messages and owning or being willing to use a promotional item was associated with smoking susceptibility; 42.2% of participants had seen tobacco sponsorship

Sirichotiratana et al. 2008	Peer-reviewed	All ASEAN countries excluding Brunei Darussalam	2001 (Singapore and Myanmar), 2003 (Cambodia, Lao PDR, Malaysia, Vietnam), 2004 (Philippines), 2005 (Thailand), 2006 (Indonesia)	Quantitative	School children aged 13-15 years in all ASEAN countries, excluding Brunei Darussalam	Proportion of participants who recalled tobacco advertising on billboards was 85.1% in Cambodia, 93.3% in Indonesia, 61.2% in Lao PDR, 84.9% in Malaysia, 75.6% in Myanmar, 87.9% in the Philippines, and 75.5% in Vietnam
Tun et al. 2017	Peer-reviewed	Myanmar	2016	Quantitative	School children aged 13-15 years (n=2,621)	83.4% of participants had noticed someone using tobacco on television, videos or movies, with males (85.3%) more likely than females (81.8%) to recall this; 42.3% of participants had noticed tobacco advertising at POS, with males (47.3%) more likely than females (37.0%) to recall this

© 2021 Stubbs T.
